# Effect of Chemical Profile on In Vitro Biological Activities of Essential Oils from Southeast Moroccan *Cladanthus eriolepis*, *Asteriscus graveolens*, and *Teucrium luteum* subsp. *flavovirens*

**DOI:** 10.3390/ijms27093983

**Published:** 2026-04-29

**Authors:** Mohamed Ouknin, El Mustapha Chibane, Hassan Alahyane, Karima Azekour, Naima Ait Aabd, Said Labbassi, Jean Costa, Lhou Majidi

**Affiliations:** 1 Regional Center of Agricultural Research of Agadir, National Institute of Agricultural Research (INRA), Avenue Ennasr, BP415 Rabat Principale, Rabat 10090, Morocco; naima.aitaabd@inra.ma (N.A.A.); said.labbassi@ced.uca.ma (S.L.); 2 Laboratory of Nanotechnology, Materials and Environment, Department of Chemistry, Faculty of Science, University Mohammed V, Rabat 10106, Morocco; sijilmassa.moss@gmail.com; 3 High Institute of Nursing Professions and Health Techniques, Beni Mellal 23000, Morocco; alahyanerh@gmail.com (H.A.); azekourkarima@yahoo.fr (K.A.); 4 Biochemistry and Integrative for Health and Environment Laboratory, Department of Biology, Polydisciplinary Faculty, Sultan Moulay Slimane University, Beni Mellal 23000, Morocco; 5 Laboratory of Agrobiotechnology and Bioengineering, Department of Biology, Faculty of Science and Technology-Gueliz, Cadi Ayyad University, Marrakesh 40000, Morocco; 6 Laboratory of Chemistry of Natural Products, Sciences and Technics Faculty, University of Corsica, 20250 Corte, France; costa_d@univ-corse.fr

**Keywords:** *Cladanthus eriolepis*, *Asteriscus graveolens*, *Teucrium luteum* subsp. *flavovirens*, essential oils, chemical composition, biological activities

## Abstract

This study aimed to elucidate the relationship between chemical profiles and in vitro biological activities of essential oils from *Cladanthus eriolepis*, *Asteriscus graveolens*, and *Teucrium luteum* subsp. *flavovirens*. The chemical composition of these essential oils has been previously reported in our recent publications, revealing distinct chemical profiles with pronounced interspecific variability and identification rates of 83.3%, 96.7%, and 98.1%, respectively. *C. eriolepis* exhibited a chemical profile rich in aliphatic esters with a mixed monoterpene–sesquiterpene composition dominated by α-pinene (13.0%), isobutyl angelate (10.7%), and 2-methylbutyl angelate (9.5%), whereas *A. graveolens* was characterized by a high abundance of 6-oxocyclonerolidol (72.5%). *T. luteum* subsp. *flavovirens* showed greater chemical complexity, including elemol (16.4%), α-pinene (12.0%), and eudesmol isomers (14.3%). All essential oils exhibited significant biological activities across various in vitro assays. *C. eriolepis* showed the strongest acaricidal effect (LC_50_ = 0.539 µL/mL) and notable inhibitory activities against acetylcholinesterase (AChE), tyrosinase, and α-glucosidase (78.4%, 74.6%, and 69.2%, respectively). *T. luteum* subsp. *flavovirens* displayed the highest antioxidant capacity (DPPH IC_50_ = 51.60 µg/mL; FRAP IC_50_ = 35.53 µg/mL). Overall, variations in chemical profiles strongly influence biological activities, highlighting their potential as multifunctional bioactive sources.

## 1. Introduction

Medicinal and aromatic plants have long been recognized as rich natural reservoirs of biologically active compounds, notably essential oils, phenolics, and flavonoids [1]. Essential oils play an important role in plant defense mechanisms [2] and have attracted considerable scientific interest due to their wide range of pharmacological activities [3]. In recent years, growing concerns regarding the safety and environmental impact of synthetic chemicals have reinforced interest in essential oils for therapeutic, cosmetic, and agricultural applications [4,5,6].

Southeastern Morocco, characterized by its arid climate and distinctive ecological conditions, is recognized for its rich diversity of aromatic and medicinal plants traditionally used by local communities [7,8]. Considering the botanical specificity of this region and in order to further investigate and valorize its medicinal flora, members of the Asteraceae and Lamiaceae families have attracted increasing scientific attention due to their diverse phytochemical composition and therapeutic potential. Within the Asteraceae family, *Cladanthus eriolepis* and *Asteriscus graveolens* are two representative species widely used in folk medicine to relieve digestive discomfort, respiratory disorders, and inflammatory conditions [9]. Phytochemical investigations have shown that their essential oils are mainly dominated by mono- and sesquiterpenes, compounds frequently associated with antioxidant, antimicrobial [10,11], and antifungal activities [12]. However, despite their traditional use, their potential as enzyme inhibitors remains relatively unexplored.

In parallel, *Teucrium luteum* subsp. *flavovirens*, a species belonging to the Lamiaceae family, is a medicinal plant traditionally used for its antiseptic and digestive properties. Species of the genus *Teucrium* are widely recognized for their richness in terpenoids and phenolic constituents, which exert a wide range of biological activities [13,14]. Although several *Teucrium* species have been investigated for their pharmacological activities [15], information regarding the enzyme inhibitory properties of *T. luteum* subsp. *flavovirens* remains limited, particularly when compared with more extensively studied species within the genus.

Beyond their phytochemical composition, the mineral profile of medicinal plants significantly contributes to their biological and nutritional value. Minerals are essential for key physiological functions, including tissue formation, metabolic regulation, and the maintenance of physiological balance. Macrolements such as calcium (Ca), phosphorus (P), magnesium (Mg), sulfur (S), potassium (K), and sodium (Na) support vital processes, while trace elements like copper (Cu) and zinc (Zn) offer health-promoting effects, e.g., potassium regulates blood pressure, magnesium aids muscle relaxation, and calcium and zinc support bone health and antioxidant defenses. Evaluating mineral composition thus provides insights into the nutritional and therapeutic potential of plants. In this context, the mineral richness of *Cladanthus eriolepis*, *Asteriscus graveolens*, and *Teucrium luteum* subsp. *flavovirens*, alongside their phytochemical constituents, enhance their medicinal and nutritional value. Mineral content can also vary with environmental factors such as soil, climate, and geographical origin, highlighting the need for comprehensive mineralogical analysis to support their valorization in traditional and modern health applications [16,17,18,19].

The therapeutic potential of the above-mentioned species remains largely unexplored, particularly with respect to their enzyme-inhibitory properties. Recently, the search for natural enzyme inhibitors has intensified due to their relevance in the management of chronic diseases, and essential oils have emerged as promising bioactive agents in this context [20,21]. Many constituents of essential oils, especially mono- and sesquiterpenes, have shown significant inhibitory effects against key metabolic and neurological enzymes [22,23].

Acetylcholinesterase (AChE) inhibition represents a key therapeutic strategy in the treatment of neurodegenerative disorders such as Alzheimer’s and Parkinson’s diseases, as it helps preserve acetylcholine levels in the central nervous system [24]. Tyrosinase, a key enzyme involved in melanin biosynthesis, is associated with hyperpigmentation disorders when overexpressed, prompting the search for safer natural alternatives to synthetic inhibitors used in cosmetic formulations. Similarly, the inhibition of α-glucosidase, an enzyme involved in carbohydrate digestion, constitutes an effective approach to attenuating postprandial hyperglycemia in patients with type 2 diabetes [25]. In this context, essential oils and other plant-derived inhibitors are increasingly considered attractive candidates due to their perceived safety and multifunctional biological activities.

Beyond their pharmacological properties, several plant-derived compounds, including essential oils, have demonstrated significant acaricidal activity [26,27,28]. Numerous studies have reported both lethal and sublethal effects of essential oils derived from Asteraceae and Lamiaceae species against mites. Essential oils from Lamiaceae species such as *Rosmarinus officinalis* L., *Lavandula angustifolia* Mill., and *Thymus vulgaris* L. have shown high efficacy in reducing mite populations when applied in different formulations [29,30]. In addition, the essential oils of these species have been reported to significantly reduce the fecundity and fertility of *Tetranychus cinnabarinus* Boisduval females [31].

Regarding Asteraceae species, several studies have reported that essential oils extracted from *Artemisia absinthium* L. and *Tanacetum vulgare* L. exert lethal effects on *Tetranychus urticae* Koch with varying degrees of efficacy. Similarly, essential oils from *Artemisia annua* L. have exhibited both lethal and sublethal activities against *Tetranychus urticae*, leading to significant reductions in fecundity, generation time, and adult longevity compared with the control [32]. Despite the rich diversity of Moroccan medicinal plants, ranked among the most diverse in the Mediterranean region, particularly within the Asteraceae and Lamiaceae families, the acaricidal properties of many species remain insufficiently explored.

This work aims to complement and extend our previous research on *Cladanthus eriolepis*, *Asteriscus graveolens*, and *Teucrium luteum* subsp. *flavovirens* by examining the behavior of their essential oils in relation to biological activity and providing a descriptive analysis of their mineral content. Biological activities were evaluated, including enzyme inhibition (acetylcholinesterase, tyrosinase, and α-glucosidase), antioxidant capacity, and acaricidal effects, with correlations applied to essential oils, offering insights into their bioactive potential and valorization prospects.

## 2. Results

### 2.1. Chemical Composition of Essential Oils

**Figure 1:**
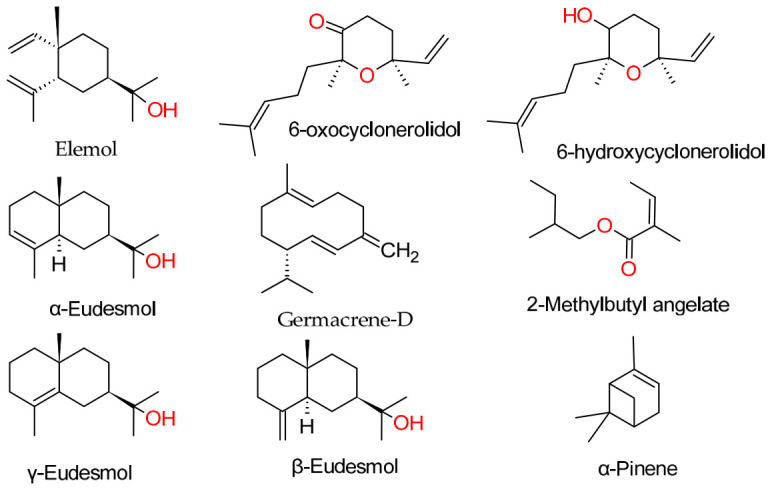
Main compounds of the studied essential oils.

In *Cladanthus eriolepis* (essential oil yield: 0.55 ± 0.09% *w*/*w*), GC–MS analysis identified 83.3% of the total oil, revealing the major chemical constituents of the essential oil. The chemical constituents of *C. eriolepis* essential oil were dominated by α-pinene (13.0%), together with a high proportion of ester compounds, mainly isobutyl angelate (10.7%) and 2-methylbutyl angelate (9.5%), which strongly shape the aromatic profile of the essential oil. Other notable chemical constituents included germacrene D (7.1%), sabinene (4.4%), β-pinene (2.5%), spathulenol (2.5%), β-bisabolene (2.4%), and 2-methylbutyl isobutyrate (2.1%), while the remaining compounds occurred at concentrations below 2% [12]. This distribution reflects a mixed monoterpene–sesquiterpene oil with a distinctive contribution from aliphatic esters (Figure 1).

The essential oil of *Asteriscus graveolens* (essential oil yield: 0.62 ± 0.10% *w*/*w*) showed a highly simplified profile, with 96.7% of the constituents identified. The oil was almost entirely composed of oxygenated sesquiterpenes, dominated by 6-oxocyclonerolidol (72.5%), followed by 6-hydroxycyclonerolidol (9.9%) and intermodeol (2.5%). Minor components included cis-chrysanthenyl acetate (2.1%), humulene epoxide II (1.3%), kessane (0.5%), (Z)-nerolidol (0.4%), and τ-cadinol (0.3%), while monoterpenes such as α-pinene (1.2%) and camphor (0.6%) were present only in low amounts. This composition clearly defines a highly specific oxygenated sesquiterpene chemotype (Table 1, Figure 1).

In contrast, the essential oil of *Teucrium luteum* subsp. *flavovirens* (essential oil yield: 0.75 ± 0.10% *w*/*w*) displayed a much more complex profile, with 98.1% of the oil identified. The major constituents were elemol (16.4%), α-pinene (12.0%), trans-caryophyllene (7.0%), α-humulene (6.4%), γ-eudesmol (5.2%), valerianol (4.9%), α-eudesmol (4.8%), and β-eudesmol (4.3%). Additional compounds such as β-pinene (5.7%), 7-epi-α-selinene (3.6%), caryophyllene oxide (2.2%), β-selinene (2.2%), and α-bisabolol (2.3%) further contributed to the essential oil complexity. This profile indicates a sesquiterpene-rich essential oil with a high proportion of oxygenated derivatives (Table 2).

### 2.2. Mineral Composition

The mineral profiles of *Cladanthus eriolepis*, *Asteriscus graveolens*, and *Teucrium luteum* subsp. *flavovirens* show marked interspecific variability, reflecting differences in mineral uptake, accumulation capacity, and possibly ecological adaptation.

Overall, macroelements (K, Ca, Mg, P, S, Na, and Cl) were present at much higher concentrations than trace elements, as expected for aerial plant parts. Among the three species, *T. luteum* subsp. *flavovirens* exhibited the highest levels of most major minerals, particularly potassium (233.88 mg/kg), calcium (244.71 mg/kg), magnesium (67.07 mg/kg), and sulfur (36.76 mg/kg), indicating a strong capacity for mineral accumulation and suggesting a higher nutritional potential. *A. graveolens* showed intermediate values, whereas *C. eriolepis* generally contained lower concentrations of these macronutrients, except for calcium, which remained relatively high (194.97 mg/kg) (Table 3).

Regarding essential trace elements, iron was the most abundant micronutrient in all species, with a pronounced enrichment in *T. luteum* subsp. *flavovirens* (56.31 mg/kg), nearly twice that observed in *C. eriolepis*. Zinc also followed a similar trend, increasing from *C. eriolepis* (0.21 mg/kg) to *A. graveolens* (0.41 mg/kg) and reaching the highest level in *T. luteum* subsp. *flavovirens* (0.54 mg/kg). Manganese, copper, cobalt, and molybdenum were detected at relatively low but comparable levels across species, suggesting a conserved physiological requirement.

Halogens such as fluorine and iodine displayed species-specific patterns: *C. eriolepis* was richer in fluorine, while iodine content was highest in *T. luteum* subsp. *flavovirens*. Elements like vanadium and silicon were notably higher in *T. luteum* subsp. *flavovirens*, which may be linked to structural or stress-related functions.

Concerning potentially toxic elements, lead (Pb) concentrations remained low in all species (≤0.18 mg/kg), indicating no apparent contamination risk. Aluminum and tin were also detected at low to moderate levels, with *T. luteum* subsp. *flavovirens* showing slightly higher aluminum content.

In summary, the table highlights clear mineral differentiation among the studied species, with *T. luteum* subsp. *flavovirens* standing out as the richest in both macro- and several micronutrients, followed by *A. graveolens*, while *C. eriolepis* exhibited comparatively lower mineral accumulation. These differences may influence their nutritional value, physiological performance, and potential applications in food, medicinal, or agro-industrial contexts.

### 2.3. Biological Activities

#### 2.3.1. Toxicity Effect

The acaricidal activities of the evaluated essential oils (EOs) are summarized in Table 4. The toxicity toward *T. urticae* adults varied considerably among the tested essential oils. Probit analysis indicated that *C. eriolepis* exhibited the highest acaricidal efficacy after 48 h of exposure, with LC_50_ and LC_90_ values of 0.539 and 6.121 µL/mL, respectively. This was followed by *T. luteum* subsp. *flavovirens* (LC_50_ = 1.482 µL/mL; LC_90_ = 9.901 µL/mL). In contrast, *A. graveolens* showed comparatively lower toxicity, with LC_50_ and LC_90_ values of 2.843 and 15.983 µL/mL, respectively.

#### 2.3.2. Phyotoxicity

Bean plants treated with the highest concentration of studied essential oils showed no signs of phytotoxicity on the leaves at any of the three evaluation times (24, 48, and 72 h). These findings indicate that the essential oils can be safely used for the management of *T. urticae* without causing damage to plant foliage.

### 2.4. Antioxidant Properties

The DPPH assay showed clear variations in radical scavenging capacity among the tested essential oils and reference compounds. *T. luteum* subsp. *flavovirens* exhibited the highest activity among the essential oils, with an IC_50_ of 51.60 µg/mL, indicating a strong ability to quench DPPH radicals. This activity was significantly higher than that of *A. graveolens* (124.68 µg/mL) and *C. eriolepis* (155.48 µg/mL) (*p* < 0.05). As expected, ascorbic acid demonstrated the most potent scavenging effect overall, with the lowest IC_50_ value (23.60 µg/mL), while BHT showed intermediate activity (104.73 µg/mL). These results highlight substantial differences in antioxidant potential depending on plant species (Table 5).

Significant differences were also observed in the FRAP assay. Ascorbic acid displayed the strongest reducing power, with an IC_50_ of 12.12 µg/mL. Among the essential oils, *T. luteum* subsp. *flavovirens* showed the most pronounced ferric-reducing capacity (35.53 µg/mL), with values close to those obtained for BHT (34.22 µg/mL). *A. graveolens* exhibited a moderate reducing effect (60.93 µg/mL), whereas *C. eriolepis* showed the weakest activity in this assay (74.97 µg/mL). These findings suggest that the antioxidant compounds present in *T. luteum* subsp. *flavovirens* are particularly effective in electron-transfer reactions.

In contrast to the DPPH and FRAP assays, a different pattern emerged in the β-carotene bleaching test. *C. eriolepis* demonstrated the strongest inhibition of lipid peroxidation among the essential oils, with an IC_50_ value of 42.88 µg/mL, outperforming both *A. graveolens* (77.47 µg/mL) and *T. luteum* subsp. *flavovirens* (121.50 µg/mL). Ascorbic acid showed moderate activity (54.57 µg/mL), while BHT exhibited a comparable but weaker effect (77.93 µg/mL). These results indicate that *C. eriolepis* may contain lipophilic antioxidant constituents that are particularly effective in protecting against lipid oxidation (Table 5).

### 2.5. Enzyme Inhibitory Activities

The inhibitory effects of the essential oils from *Cladanthus eriolepis*, *Asteriscus graveolens*, and *Teucrium luteum* subsp. *flavovirens* against acetylcholinesterase (AChE), tyrosinase, and α-glucosidase were evaluated in vitro at increasing concentrations, and the corresponding inhibition percentages are summarized in Table 6. Overall, all tested essential oils exhibited a clear concentration-dependent inhibitory response, with enzyme inhibition becoming more pronounced at higher doses. According to the classification proposed by Custódio et al. [35], inhibition values above 50% are considered potent, indicating that most of the tested concentrations, particularly at 0.8 and 1.2 mg/mL, displayed strong enzymatic inhibition.

Regarding AChE inhibition, all three essential oils showed potent activity across the tested concentration range (0.2, 0.4, 0.8, and 1.2 mg/mL). *C. eriolepis* exhibited the highest inhibition, reaching 97.33% at 1.2 mg/mL, which exceeded the activity of the positive control. Similarly, *A. graveolens* and *T. luteum* subsp. *flavovirens* demonstrated strong AChE inhibitory effects at the highest concentration (93.50%), confirming a strong positive correlation between concentration and inhibitory efficiency.

Tyrosinase inhibition also increased significantly with concentration for all essential oils. *C. eriolepis* displayed the most pronounced activity, achieving 87.43% inhibition at 1.2 mg/mL, followed closely by *A. graveolens* (85.63%) and *T. luteum* subsp. *flavovirens* (81.37%). At higher concentrations, the inhibitory effects of these essential oils were comparable to or slightly exceeded those of the positive control, indicating a strong anti-tyrosinase potential.

In the case of α-glucosidase, the essential oils showed marked inhibitory activity, particularly at elevated concentrations. *C. eriolepis* exhibited the strongest effect, with inhibition reaching 89.62% at 1.2 mg/mL, surpassing the reference inhibitor. *A. graveolens* also demonstrated high inhibition (86.87%), closely matching the positive control, while *T. luteum* subsp. *flavovirens* showed slightly lower, yet still potent, inhibition (82.03%). The strong inhibitory effects observed, especially at higher doses, may be attributed to the major bioactive constituents of the essential oils or to possible synergistic interactions among their components (Table 6).

### 2.6. Correlation Matrix

**Figure 2:**
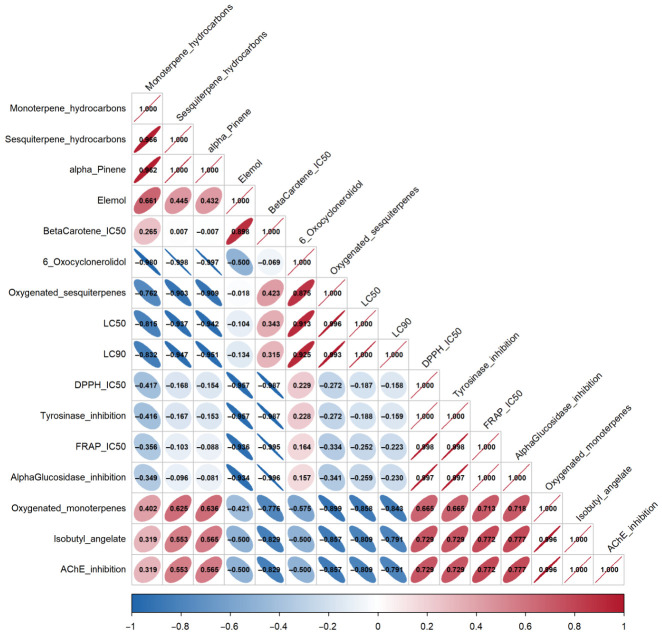
Correlation matrix between chemical composition variables (terpene classes and major compounds) and biological activities (acaricidal, antioxidant, and enzyme inhibition assays) of the essential oils of *Cladanthus eriolepis*, *Asteriscus graveolens*, and *Teucrium luteum* subsp. *flavovirens*. Values are presented as descriptive/exploratory trends (*n* = 3).

Pearson r values between chemical composition variables and biological activities were computed to describe compositional–activity trends across the three essential oils (*n*  =  3); given this sample size, these values are presented as exploratory trends only (Figure 2). The ratio of hydrocarbon to oxygenated terpene content emerged as the principal factor linking chemical class composition to activity profiles. For acaricidal activity, oxygenated sesquiterpene content showed the strongest positive associations with LC_50_ (r = 0.996) and LC_90_ (r = 0.993), while sesquiterpene hydrocarbon content (r = −0.937 and −0.947) and α-pinene content (r = −0.942 and −0.951) showed the strongest negative associations. The major compound of *A. graveolens*, 6-oxocyclonerolidol, showed positive associations with LC_50_ (r = 0.913) and LC_90_ (r = 0.925), consistent with the lowest acaricidal potency recorded for this species.

For antioxidant activity, elemol showed marked negative associations with DPPH IC_50_ (r = −0.957) and FRAP IC_50_ (r = −0.936), and a positive association with β-carotene bleaching IC_50_ (r = 0.898), consistent with the polar antioxidant profile of *T. luteum*, the only species containing this compound (16.4%). Oxygenated monoterpene content showed moderate positive associations with DPPH IC_50_ (r = 0.665) and FRAP IC_50_ (r = 0.713), and a negative association with β-carotene bleaching IC_50_ (r = −0.776).

For enzyme inhibition, isobutyl angelate and oxygenated monoterpene content showed the strongest positive associations with AChE inhibition (r = 1.000 and r = 0.996, respectively), while oxygenated monoterpene content also showed a positive association with α-glucosidase inhibition (r = 0.718). Oxygenated sesquiterpene content showed a negative association with AChE inhibition (r = −0.857). Overall, higher hydrocarbon terpene content was linked to stronger acaricidal and polar antioxidant activity, whereas higher oxygenated monoterpene content was associated with greater enzyme inhibition.

### 2.7. Radar Chart Analysis

**Figure 3:**
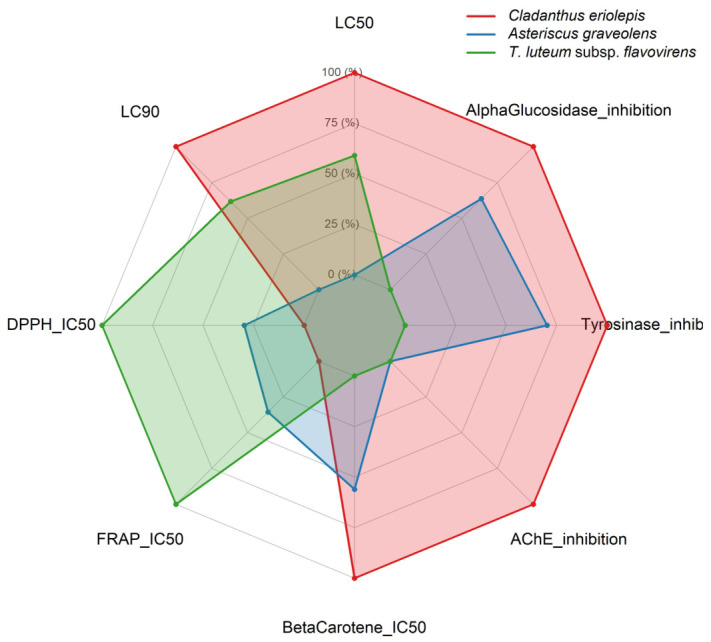
Radar chart comparing the normalized biological activities of the essential oils of *Cladanthus eriolepis*, *Asteriscus graveolens*, and *Teucrium luteum* subsp. *flavovirens*.

Radar chart analysis of the eight bioassays revealed distinct activity profiles among the three essential oils, with *C. eriolepis* recording the highest normalized values in six of the eight assays (Figure 3). This species exhibited the lowest LC_50_ (0.539 µL/mL) and LC_90_ (6.121 µL/mL) in the acaricidal bioassay against *Tetranychus urticae*, the lowest β-carotene bleaching IC_50_ (42.88 µg/mL), and the highest enzyme inhibition at 1.2 mg/mL across all three targets (AChE: 97.33%; tyrosinase: 87.43%; α-glucosidase: 89.62%). *T. luteum* recorded the lowest DPPH IC_50_ (51.60 µg/mL) and FRAP IC_50_ (35.53 µg/mL). *A. graveolens* did not record the highest normalized value in any assay, showing intermediate enzyme inhibition and the lowest acaricidal potency (LC_50_ = 2.843 µL/mL). *C. eriolepis* and *T. luteum* thus displayed complementary activity profiles, each leading in a distinct functional domain, while *A. graveolens* occupied an intermediate position across all assays.

**Figure 4:**
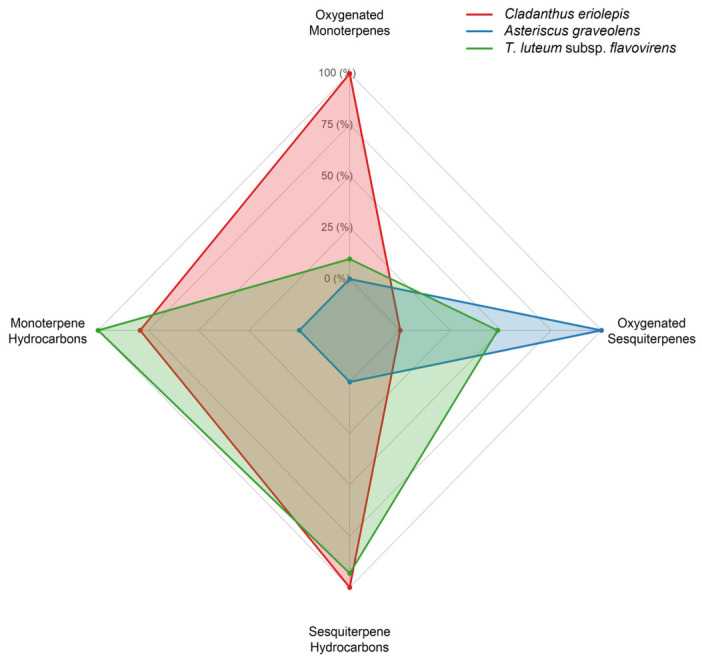
Radar chart comparing the chemical class composition (monoterpene hydrocarbons, sesquiterpene hydrocarbons, oxygenated monoterpenes, and oxygenated sesquiterpenes) of the essential oils of *Cladanthus eriolepis*, *Asteriscus graveolens*, and *Teucrium luteum* subsp. *flavovirens*.

The chemical class radar chart revealed clear differences in terpene class distribution among the three essential oils (Figure 4). *T. luteum* had the highest total hydrocarbon terpene content (monoterpene hydrocarbons and sesquiterpene hydrocarbons = 42.3%), with low oxygenated monoterpenes (7.5%) and moderate oxygenated sesquiterpenes (48.3%). *C. eriolepis* had the highest oxygenated monoterpene content (34.2%), high sesquiterpene hydrocarbons (23.7%), moderate monoterpene hydrocarbons (16.3%), and the lowest oxygenated sesquiterpenes (9.1%). *A. graveolens* showed a markedly distinct pattern, with negligible hydrocarbons (1.5% monoterpene hydrocarbons; 0.6% sesquiterpene hydrocarbons) and 90.0% oxygenated sesquiterpenes.

## 3. Discussion

### 3.1. Chemical Composition

The mean essential oil yield obtained by hydrodistillation from *Teucrium luteum* subsp. *flavovirens* was 0.75%, which is substantially higher than the yields reported for populations collected in Monastir, Tunisia (0.02%) [13] and Jebel Aïssa, Algeria (0.42%) [14]. For *Asteriscus graveolens*, an essential oil yield of 0.62% was recorded, differing from those reported for samples collected in Tamanrasset, southern Algeria (0.30%), and South El-Dahnaa, Saudi Arabia (0.61%) [36]. Likewise, the essential oil yield from the aerial parts of *Cladanthus eriolepis* reached 0.55%, which contrasts with the lower yield reported for material collected in Ijoukak, High Atlas, Morocco (0.37%) [37]. Overall, essential oil yields exhibited pronounced variability across sampling sites, underscoring the strong influence of geographical origin and local environmental conditions on essential oil productivity. This variability is commonly attributed to differences in climatic factors, soil properties, altitude, and phenological stage, as consistently documented in previous studies [5,6,38,39].

The essential oils of the three studied species displayed distinct terpene-dominated chemical profiles. *Cladanthus eriolepis* essential oil was mainly composed of oxygenated monoterpenes (34.2%) and sesquiterpene hydrocarbons (23.7%), with α-pinene (13.0%), isobutyl angelate (10.7%), 2-methylbutyl angelate (9.5%), and germacrene D (7.1%) as the major constituents, whereas populations from the High Atlas of Morocco were richer in sabinene (15.6–23.8%), α-pinene (12.2–17.7%), α-phellandrene (7.1%), terpinen-4-ol (4.6–11.0%), germacrene D (2.7–5.7%), and borneol (0.4–6.2%) [37], and another population was dominated by camphor (37.02%), followed by sabinene (10.26%), α-pinene (6.29%), p-cymene (6.13%), and α-cadinol (5.57%) [10]. *Asteriscus graveolens* essential oil was overwhelmingly rich in oxygenated sesquiterpenes (90.0%), mainly 6-oxocyclonerolidol (72.5%), 6-hydroxycyclonerolidol (9.9%), and intermodeol (2.5%), while other populations showed distinct compositions such as α-thujone (17.92%), carvacrol (14.14%), p-cineole (13.83%), and camphor (12.71%) in South El-Dahnaa, Saudi Arabia [36], or cis-8-acetoxychrysanthenyl acetate (48.6%), epi-α-muurolol (14.5%), β-eudesmol (6.2%), and chrysanthenyl acetate (5.2%) [40]. Finally, *Teucrium luteum* subsp. *flavovirens* exhibited a sesquiterpene-rich profile, with oxygenated sesquiterpenes (48.3%) and sesquiterpene hydrocarbons (22.1%), mainly elemol (16.4%), trans-caryophyllene (7.0%), α-humulene (6.4%), γ-eudesmol (5.2%), valerianol (4.9%), α-eudesmol (4.8%), β-elemol (7.2%), (+)-α-pinene (6.0%), β-eudesmol (5.5%), guaiol (4.2%), α-bisabolol (4.2%), and β-caryophyllene (4.1%), consistent with populations from Monastir, Tunisia [13]. The observed differences in the chemical composition of the essential oils, both in our study and in previous reports, are likely linked to soil characteristics and geographical conditions of the areas where the plants were harvested [41,42].

The mineral analysis of the aerial parts of *C. eriolepis*, *A. graveolens*, and *T. luteum* subsp. *flavovirens* revealed distinct profiles of essential macro- and micronutrients. Notably, *T. luteum* exhibited the highest levels of potassium (233.88 ± 4.71 mg/kg), calcium (244.71 ± 9.81 mg/kg), magnesium (67.07 ± 2.12 mg/kg), and iron (56.31 ± 1.31 mg/kg), whereas *A. graveolens* and *C. eriolepis* showed moderate to lower concentrations of these elements. Trace elements such as zinc, molybdenum, iodine, and vanadium were present in measurable amounts, while potentially toxic elements like lead remained very low (≤0.18 mg/kg). These variations are likely influenced by soil composition and environmental conditions, consistent with trends observed in other medicinal and aromatic plants [43,44,45]. Importantly, the concentrations of all minerals are within the safety limits established by the World Health Organization (WHO, 1998) [46], supporting the potential use of these species in dietary supplements, herbal remedies, and medicinal applications.

### 3.2. Biological Activity Profiles

The antioxidant assays revealed significant activity in all examined essential oils across three methods: DPPH, FRAP, and β-carotene bleaching. The oils’ activity appears closely related to their chemical composition, particularly the abundance of oxygenated sesquiterpenes and monoterpenes. For example, the complex essential oil of *Teucrium luteum* subsp. *flavovirens*, rich in elemol (16.4%), α-pinene (12.0%), trans-caryophyllene (7.0%), and α-humulene (6.4%), showed the strongest radical scavenging activity in DPPH (IC_50_ = 51.60 ± 1.87 µg/mL) and FRAP assays (IC_50_ = 35.53 ± 1.05 µg/mL), approaching the activity of the synthetic antioxidant BHT. In contrast, *Asteriscus graveolens*, dominated by oxygenated sesquiterpenes such as 6-oxocyclonerolidol (72.5%), displayed moderate activity (DPPH IC_50_ = 124.68 ± 3.76 µg/mL; FRAP IC_50_ = 60.93 ± 4.41 µg/mL), while *Cladanthus eriolepis*, with a mixed monoterpene–sesquiterpene composition including α-pinene (13.0%) and isobutyl angelate (10.7%), exhibited the lowest radical scavenging effect (DPPH IC_50_ = 155.48 ± 4.83 µg/mL; FRAP IC_50_ = 74.97 ± 4.19 µg/mL). The essential oils of *T. luteum* subsp. *flavovirens* collected from the Monastir region are characterized by a diverse chemical profile, notably containing β-elemol (7.2%), (+)-α-pinene (6.0%), β-eudesmol (5.5%), guaiol and α-bisabolol (4.2% each), along with β-caryophyllene (4.1%). This composition is associated with a pronounced antioxidant capacity, as demonstrated by the DPPH radical scavenging assay, which yielded an IC_50_ value of 8.39 ± 0.24 µg/mL, indicating strong free radical–quenching activity [13]. The essential oils of *Teucrium gypsophilum* exhibited significant antioxidant activity, with a FRAP value corresponding to an IC_50_ of 63.53 ± 2.50 mg TE/g. They are primarily composed of β-pinene (28.6%), diethyl phthalate (20.7%), spathulenol (10.49%), ledol (6.92%), germacrene D (6.04%), α-pinene (5.8%), and D-limonene (4.26%), reflecting the strong ferric-reducing capacity of their major constituents [47].

Interestingly, *C. eriolepis* exhibited the strongest inhibition of lipid peroxidation in the β-carotene bleaching assay (IC_50_ = 42.88 ± 2.43 µg/mL). This pronounced antioxidant activity is likely related to its distinctive chemical profile, characterized by a predominance of monoterpene hydrocarbons and ester derivatives, mainly α-pinene (13.0%), isobutyl angelate (10.7%), and 2-methylbutyl angelate (9.5%). Additional contributions from germacrene D (7.1%), sabinene (4.4%), β-pinene (2.5%), spathulenol (2.5%), β-bisabolene (2.4%), and 2-methylbutyl isobutyrate (2.1%) may also synergistically enhance its antioxidant potential. In line with these findings, Sarikurkcu et al. [48] reported that the essential oil of *Vitex agnus-castus* L., characterized by relatively high levels of sabinene (13.45%) and α-pinene (10.60%), also exhibited notable antioxidant activity in the β-carotene bleaching assay, with an inhibition percentage of 86.17% at a concentration of 2.0 mg·mL^−1^. These compounds may contribute to lipid peroxidation inhibition either through their individual antioxidant capacities or via synergistic interactions between major and minor constituents [12,49].

Our findings indicated that the lethal concentrations (LC_50_ and LC_90_) varied markedly over the tested essential oils. At 48 h after treatment, *C. eriolepis* essential oil exhibited the highest efficacy against *T. urticae* adults compared with the other evaluated EOs, with LC_50_ and LC_90_ values of 0.539 µL/mL and 6.121 µL/mL, respectively. The acaricidal activity of the studied EOs may be due to their chemical composition, particularly their major constituents. These essential oils are rich in monoterpenoids, compounds that have been extensively reported to exhibit significant acaricidal activity against various mite species [50,51]. In this regard, Ouknin et al. [28] reported that the acaricidal effects of EOs are largely associated with their major components. Conversely, studies by Wu et al. [52] and Alvares et al. [53] suggested that the acaricidal effects of essential oils cannot be attributed solely to their major constituents but may also result from synergistic interactions between the minor compounds. According to the chemical profile of *C. eriolepis* EO, isobutyl angelate and 2-methylbutyl angelate were identified as major compounds and are likely responsible for the pronounced acaricidal activity observed. Similarly, Pirali-Kheirabadi et al. [54] reported that *Matricaria chamomilla* essential oil, in which isobutyl angelate is a dominant constituent, exhibited the highest acaricidal activity against the cattle fever tick (Acari: Ixodidae). In the same context, all chamomile extracts tested showed highly significant acaricidal activity compared with the control [55].

The enzyme inhibition assays demonstrated that the essential oils of *C. eriolepis*, *A. graveolens*, and *T. luteum* subsp. *flavovirens* exerted clear concentration-dependent inhibitory effects against acetylcholinesterase (AChE), tyrosinase, and α-glucosidase, with inhibition increasing progressively across the tested concentration range (0.2–1.2 mg/mL). Among the investigated oils, *C. eriolepis* essential oil showed the strongest overall activity, achieving near-complete inhibition of AChE and high inhibition of both tyrosinase and α-glucosidase at the highest concentration, with values exceeding those of the positive controls, thereby highlighting its pronounced enzyme-targeting capacity. The essential oil of *A. graveolens* also exhibited strong inhibitory effects, particularly against tyrosinase and α-glucosidase, where its activity at higher concentrations closely approached that of the reference compounds, indicating an important contribution of its volatile constituents to enzyme modulation. In comparison, *T. luteum* subsp. *flavovirens* essential oil displayed moderate to high inhibition, with a more pronounced effect on AChE than on the other enzymes, while maintaining a consistent dose–response trend. Overall, these results suggest that the observed inhibitory activities are not attributable to a single dominant compound but rather arise from the combined and potentially synergistic actions of major and minor volatile constituents, supporting the concept of essential oils as complex bioactive systems capable of interacting with multiple enzymatic targets [56,57,58]. Several studies have reported that major compounds such as α-pinene and β-caryophyllene, along with other monoterpenes and sesquiterpenes, exhibit significant inhibitory activity against acetylcholinesterase (AChE), tyrosinase, and α-glucosidase, contributing to the neuroactive and multifunctional properties of essential oils [59,60,61,62,63,64].

The activity profiles revealed by radar chart analysis (Figure 3) are consistent with the chemical class distributions of the three essential oils, suggesting that the balance between hydrocarbon and oxygenated terpenes influences the assays in which each oil exhibits the strongest activity. The dominance of *C. eriolepis* in six out of eight assays aligns with its balanced terpene composition, combining substantial fractions of both hydrocarbon and oxygenated monoterpenes, which may account for its performance in both antioxidant and enzyme inhibition assays.

Among the individual compounds identified in *C. eriolepis*, isobutyl angelate (10.7%), detected exclusively in this species, exhibited a perfect positive correlation (r value) with AChE inhibition. Angelate esters contain an α,β-unsaturated ester moiety, a structural feature associated with cytotoxic activity in other Asteraceae [65]. Acetylcholinesterase possesses a catalytic site in which three amino acids, serine, histidine, and glutamate, act cooperatively to hydrolyze acetylcholine [66]. The α,β-unsaturated ester group of isobutyl angelate may interact with these residues, potentially interfering with the catalytic activity of the enzyme [67]. In addition, the ester oxygen can form hydrogen bonds with glycine residues (Gly121 and Gly122) located near the active site, while the isobutyl chain may occupy a neighboring hydrophobic pocket formed by aromatic residues such as Trp86 and Tyr337 [68]. Together, these interactions provide a plausible molecular explanation for the strong AChE inhibition observed in *C. eriolepis* essential oil [67,68].

α-Pinene (13.0%), the most abundant monoterpene hydrocarbon, showed strong negative correlations with acaricidal LC_50_ and LC_90_ values, consistent with previously reported in vitro cytotoxic effects of this compound [69]. Germacrene D (7.1%) has been identified as a major component of essential oils that exhibit lipid peroxidation inhibition (IC_50_ = 12.78 µg/mL) [70]. Furthermore, α-glucosidase inhibition has been reported across the tribe Cardueae, with *Centaurea lycaonica* showing higher activity than acarbose [71] and *C. urvillei* exhibiting IC_50_ values around 18 µg/mL [72].

The divergence between β-carotene bleaching and DPPH/FRAP responses observed for *C. eriolepis* reflects the distinct physicochemical environments of these assays. The β-carotene bleaching test evaluates lipid peroxidation inhibition in an emulsion system, where nonpolar compounds tend to be more effective [73,74]. In contrast, DPPH and FRAP assays are conducted in polar media, where nonpolar hydrocarbons generally exhibit lower activity [75].

The strong polar antioxidant activity of *T. luteum* is consistent with the presence of elemol (16.4%), an oxygenated sesquiterpene alcohol absent from the other two oils. Essential oils rich in elemol have demonstrated notable DPPH scavenging activity, largely attributed to oxygenated sesquiterpenes [13]. The DPPH IC_50_ value of *T. luteum* was lower than that reported for *T. polium* essential oil (IC_50_ = 61.38 µg/mL) [76] and also exceeded the activity of BHT, in agreement with findings reported for a Tunisian population of the same subspecies [13]. trans-Caryophyllene (7.0%) and α-pinene (12.0%) have both demonstrated radical-scavenging activity in DPPH assays [69,77], while trans-caryophyllene has also been associated with anti-inflammatory effects [78].

The intermediate position of *A. graveolens* reflects its distinctive composition, dominated by 6-oxocyclonerolidol (72.5%) and 6-hydroxycyclonerolidol (9.9%), with only 2.1% total hydrocarbons. Although it ranked last among the three oils in terms of acaricidal activity, *A. graveolens* still exhibited enzyme inhibition at 1.2 mg/mL, comparable to that of the positive controls. Essential oils rich in 6-oxocyclonerolidol have demonstrated antifungal activity in *A. graveolens* from Morocco [33] and in *Anvillea gracinii* [79], as well as antioxidant and antiproliferative activities in *Perralderia coronopifolia* [80]. These oxygenated nerolidol derivatives may interact more effectively with enzyme active sites due to their higher polarity [78].

The terpene class profiles (Figure 4) paralleled the activity patterns described above. Hydrocarbon-rich oils (*T. luteum*, *C. eriolepis*) exhibited stronger polar antioxidant and acaricidal activities, whereas the highest content of oxygenated monoterpenes (*C. eriolepis*) was associated with the most pronounced enzyme inhibition. *A. graveolens*, dominated by oxygenated sesquiterpenes, showed intermediate responses. Hydrocarbon terpenes are nonpolar and tend to accumulate in lipid phases, where they may inhibit oxidation reactions [74,75]. In contrast, oxygenated terpenes contain functional groups (e.g., hydroxyl, carbonyl, and ester) that may facilitate specific interactions with enzyme active sites [78].

### 3.3. Exploratory Correlation Analysis

The correlation matrix analysis (Figure 2), based on three essential oils (*n* = 3), provides a descriptive framework linking chemical composition to biological activity patterns. The negative associations between hydrocarbon terpene content and acaricidal LC_50_/LC_90_ values are consistent with the higher lipophilicity of these compounds, which facilitates their penetration through arthropod cuticles and the disruption of cell membranes [81]. In contrast, the positive associations between 6-oxocyclonerolidol content and LC_50_/LC_90_ values reflect the lower acaricidal potency of the oxygenated sesquiterpene-dominated *A. graveolens* essential oil.

Elemol showed descriptive associations with DPPH and FRAP but not with β-carotene bleaching, suggesting that its antioxidant activity operates preferentially in polar solvent systems rather than in lipid emulsions [73]. This is consistent with the attribution of antioxidant activity in sesquiterpene alcohol-rich oils to oxygenated sesquiterpenoids as a class [13]. Antioxidant compounds usually neutralize free radicals by transferring a hydrogen atom from a hydroxyl (–OH) group, a process known as hydrogen atom transfer [82]. In elemol, this hydroxyl group is attached to a simple carbon chain without any aromatic ring, which makes the hydrogen atom less easily transferable than in aromatic antioxidants such as α-tocopherol (vitamin E) [83]. This explains the moderate DPPH scavenging activity of elemol, while the same hydroxyl group is still sufficient to support its reducing activity in the FRAP assay, consistent with the polar antioxidant profile of *T. luteum* [84].

The positive descriptive associations between oxygenated monoterpene content and enzyme inhibition (particularly AChE and α-glucosidase) suggest that the polar functional groups present in these compounds (hydroxyl, carbonyl, and ester) may facilitate interactions with enzyme active sites [85]. Isobutyl angelate was present exclusively in *C. eriolepis* and showed a high positive r value with AChE inhibition, indicating a possible association between this ester and the enzyme-inhibitory activity of this oil. Nevertheless, the descriptive patterns were internally consistent across chemical classes and biological assays.

## 4. Materials and Methods

### 4.1. Plant Material

The aerial portions of *Cladanthus eriolepis*, *Asteriscus graveolens*, and *Teucrium luteum* subsp. *flavovirens* were collected during their full flowering stage from several ecological regions of Morocco. The botanical identity of the samples (Table 7) was established based on the Practical Flora of Morocco [86] and later verified at the Herbarium of the Faculty of Science and Technologies, Errachidia. After collection, the plant materials were air-dried under ambient laboratory conditions. For each species, essential oils were extracted from 100 g of dried plant material through hydrodistillation using a Clevenger-type apparatus for a period of three hours in accordance with the procedure described in the European Pharmacopoeia (1997) [87]. The obtained oils were subsequently dried over anhydrous sodium sulfate, filtered to remove residual moisture, and stored at −4 °C until further analysis.

### 4.2. Plant Mineral Analysis

The aerial parts of each plant species were treated individually following a standardized preparation procedure. Initially, the samples were thoroughly rinsed with distilled water and then dried in an oven (Memmert GmbH + Co. KG, Schwabach, Germany) at 80 °C until a constant mass was obtained. The dried plant material was subsequently ground to a fine powder, and a portion of 0.5 g was used for the mineralization process. This sample was digested with an acid mixture composed of 2 mL of concentrated sulfuric acid (98%), 6 mL of nitric acid (65%), and 6 mL of hydrogen peroxide (35%). Digestion was carried out on a sand bath for approximately 30 min to ensure complete degradation of the plant matrix. After allowing the digest to cool, the solution was filtered and diluted to a final volume of 25 mL using 0.1 M nitric acid [43]. The concentrations of mineral elements in the prepared solutions were subsequently determined by inductively coupled plasma optical emission spectrometry (ICP-OES) using JOBIN-YVON 70 ULTIMA and JY 70 instruments, developed by Horiba Scientific (formerly Jobin-Yvon, Palaiseau, 16–18 rue du Canal, Longjumeau Cedex, France).

### 4.3. GC and GC-MS Analysis

Essential oil composition was determined using a Perkin-Elmer Autosystem XL gas chromatograph (GC) (PerkinElmer, Inc., 940 Winter Street, Waltham, MA, USA) equipped with dual flame ionization detectors (FID) and two fused-silica capillary columns, Rtx-1 (polydimethylsiloxane) and Rtx-Wax (polyethylene glycol), each measuring 60 m in length, 0.22 mm in internal diameter, and with a film thickness of 0.25 µm. The oven temperature was initially set at 60 °C and increased to 230 °C at a rate of 2 °C min^−1^, followed by a 35 min isothermal period at 230 °C, while both the injector and detector temperatures were maintained at 280 °C. For GC-FID analysis, 0.2 µL of undiluted essential oil was injected in split mode (1:50) using helium as the carrier gas at a constant flow rate of 1 mL min^−1^. Retention indices (RI) were calculated relative to a homologous series of n-alkanes (C5–C30) according to the equation of Van den Dool and Kratz [88], and the relative abundance of each component was estimated from GC peak areas without applying correction factors. The same samples were subsequently analyzed by gas chromatography–mass spectrometry (GC–MS) using a Perkin-Elmer Autosystem XL GC coupled with a Perkin-Elmer Turbo mass detector (quadrupole), operating under identical chromatographic conditions and column specifications. Helium served as the carrier gas at 1 mL min^−1^, the injector temperature was maintained at 280 °C, and the ion source temperature was set at 150 °C. Mass spectra were obtained by electron ionization (EI) at 70 eV over a mass range of 35–350 amu, with injections performed in split mode (1:80) using 0.2 µL of pure essential oil.

### 4.4. Biological Activities

#### 4.4.1. Toxicity Tests

The acaricidal effects of essential oils (EOs) on *T. urticae* adults were assessed following the method of Miresmailli et al. [89], with slight modifications. Leaf discs (4 cm in diameter) were treated with 100 µL of each concentration of the three tested EOs (0.125, 0.25, 0.5, 1, and 2 µL/mL) or with the control solution. Treated discs were allowed to dry for 5 min under laboratory conditions before being placed in 9 cm diameter Petri dishes containing moistened cotton. Twenty *T. urticae* adults of the same age were transferred onto the treated leaf discs using a fine brush. Each treatment was replicated three times. Mortality of *T. urticae* was recorded 48 h post-treatment, and the number of eggs deposited on the leaf discs was also counted. Individuals were considered dead if no movement of appendages was observed when gently prodded with a fine brush.

#### 4.4.2. Phyotoxicity

The phytotoxicity effects of the studied essential oils (EOs) on bean plants were assessed according to the method used by Ouknin et al. [27]. Bean plants were treated with the highest concentration (2%) of each EO. The entire leaf surface was uniformly sprayed with 10 mL of each EO solution or the control without EOs using a hand sprayer. Treated plants were maintained at 24 ± 2 °C, 65 ± 5% relative humidity, and a 16:8 h light–dark photoperiod. Phytotoxicity was evaluated at 24, 48, and 72 h post-treatment and classified into six levels, ranging from grade 0 (no visible injury) to grade 5 (complete leaf necrosis).

### 4.5. Antioxidant Activities

#### 4.5.1. DPPH Assay

The antioxidant activity of the essential oils was assessed using the DPPH (2,2-diphenyl-1-picrylhydrazyl) free radical scavenging assay according to the method reported by Ouknin et al. [90]. In this test, various concentrations of the essential oils were added to a 0.4 mM methanolic DPPH solution, using a mixture ratio of 50 μL of sample to 5 mL of DPPH solution. The reaction mixtures were kept in the dark for 30 min to allow the scavenging reaction to occur, after which the absorbance was recorded at 517 nm with a Uviling 9400 spectrophotometer (SECOMAM, Aqualabo Group, Aqualabo, France). Butylated hydroxytoluene (BHT) and ascorbic acid served as reference antioxidants. The ability of the samples to neutralize DPPH radicals was expressed as the percentage of inhibition, calculated according to Equation (1):

(1) $$DPPH\;Scavenging\;effect\;\left(\%\right)=\left(\frac{A_{0}-A_{1}}{A_{0}}\right)\times 100$$

A_0_ refers to the absorbance of the control measured after 30 min, whereas A_1_ denotes the absorbance of the sample under the same conditions. All measurements were carried out in triplicate.

#### 4.5.2. Reducing Power Determination (FRAP)

The reducing power of the essential oils was determined by evaluating their ability to convert Fe^3+^ to Fe^2+^, following the method described by Oyaizu [91]. The essential oils and reference antioxidants were first diluted in ethanol to obtain concentrations ranging from 20 to 500 µg/mL for the oils and from 5 to 100 µg/mL for the standards. Each solution was mixed with 2.5 mL of phosphate buffer (0.2 M, pH 6.6) and 2.5 mL of potassium ferricyanide [K_3_Fe(CN)_6_] at 1%. The mixtures were incubated at 50 °C for 20 min, after which 2.5 mL of trichloroacetic acid (10%) was added. Following centrifugation at 3000 rpm for 10 min, 2.5 mL of the supernatant was combined with an equal volume of distilled water and 0.5 mL of FeCl_3_ (0.1%). The absorbance of the resulting solution was then recorded at 700 nm using a spectrophotometer.

The IC_50_ value, defined as the concentration of the essential oil that produces an absorbance of 0.5, was determined by plotting measured absorbance against corresponding concentrations. BHT and Ascorbic acid served as reference standards. All measurements were carried out in triplicate, and the IC_50_ values are reported as mean ± standard deviation.

#### 4.5.3. β-Carotene Bleaching Test

The antioxidant activity of the essential oils was examined using the β-carotene/linoleic acid bleaching method, following a procedure adapted from Ouknin et al. [34]. In this assay, Ascorbic acid and BHT were included as positive controls, while ethanol was used as the negative control in place of the oils. The antioxidant efficiency of the tested samples was expressed through the rate of β-carotene bleaching, calculated using Equation (2):

(2) $$I\%=\left(\frac{A_{\beta -carotene\;after\;2\;h}}{A_{initial\;\beta -carotene}}\right)\times 100$$

A_β-carotene after 2 h_ corresponds to the absorbance measured for the remaining β-carotene after two hours of incubation, while A_initial β-carotene_ refers to the absorbance recorded at the beginning of the assay. All analyses were performed in triplicate. The IC_50_ value, which represents the concentration of the essential oil needed to achieve 50 percent inhibition, was determined from a curve produced by plotting inhibition percentage against tested concentrations.

### 4.6. Enzyme Inhibitory Properties

#### 4.6.1. AChE Inhibition

The inhibitory effect of the essential oils on cholinesterase activity was evaluated using a microplate reader based on a modified method adapted from Ouknin et al. [6]. In each assay well, 25 μL of 15 mM acetylthiocholine iodide (ATCI) was mixed with 125 μL of 3 mM 5,5′-dithiobis-(2-nitrobenzoic acid) (DTNB), followed by the addition of 50 μL of phosphate buffer (100 mM, pH 8.0) and 25 μL of essential oil at different concentrations (0.2, 0.4, 0.8, and 1.2 mg/mL). For reference, galanthamine (25 μg/mL) was used as the positive control, while the buffer served as the negative control. The reaction was initiated by adding acetylcholinesterase (AChE, 0.28 U/mL), and the variation in absorbance was recorded at 405 nm for 5 min. Enzyme inhibition was calculated relative to the buffer control, and all experiments were conducted in triplicate to ensure reproducibility.

#### 4.6.2. Tyrosinase Inhibition

Tyrosinase inhibitory activity was measured using a spectrophotometric method adapted from Masuda et al. [92]. In the assay, essential oils at concentrations of 0.2, 0.4, 0.8, and 1.2 mg/mL, or phosphate buffer as a blank, were added to 80 μL of phosphate buffer (pH 6.8). This was followed by the addition of 40 μL of L-DOPA and 40 μL of tyrosinase enzyme. Kojic acid at 200 μg/mL was included as a reference standard. Absorbance was recorded at 475 nm, and the percentage of tyrosinase inhibition was calculated. All measurements were performed in triplicate.

#### 4.6.3. α-Glucosidase Inhibition

The inhibitory potential of the essential oils against α-glucosidase was evaluated according to the procedure reported by Kwon et al. [93]. In this assay, different concentrations of essential oils (0.2, 0.4, 0.8, and 1.2 mg/mL) were mixed with 50 μL of phosphate-buffered solution (0.1 M, pH 6.9) containing yeast α-glucosidase (1.0 U/mL), while acarbose (1 mg/mL) served as the positive reference inhibitor. The reaction mixtures were allowed to stand at room temperature for 10 min before the addition of 50 μL of 5 mM p-nitrophenyl-α-D-glucopyranoside as the substrate. After a further incubation period of 10 min, the absorbance was recorded at 405 nm to monitor the enzymatic reaction, and the percentage inhibition of α-glucosidase activity was subsequently calculated. All measurements were performed in triplicate to ensure accuracy and reproducibility.

### 4.7. Statistical Analysis

Statistical analyses were performed in R (v4.4.1; R Foundation for Statistical Computing, Vienna, Austria). One-way ANOVA followed by Tukey’s HSD test was used to compare group means. Pearson correlation analysis was conducted to assess relationships among variables, and results were visualized using correlation matrices and radar plots.

## 5. Conclusions

This study demonstrated that the in vitro biological activities of essential oils from *Cladanthus eriolepis, Asteriscus graveolens*, and *Teucrium luteum* subsp. *flavovirens* are closely influenced by their chemical compositions, with marked interspecific differences mainly characterized by oxygenated sesquiterpenes and monoterpenes that appear to play a key role in their bioactivities. A clear relationship was observed between the presence and relative abundance of these major constituents and the antioxidant capacity, lipid peroxidation inhibition, and enzyme-modulating activities, in addition to significant effects against *Tetranychus urticae* and strong inhibitory activities toward acetylcholinesterase, tyrosinase, and α-glucosidase, highlighting their multi-target potential. Among the studied species, *C. eriolepis* exhibited the most pronounced and broad-spectrum activity, suggesting synergistic interactions between major and minor constituents that enhance its efficacy. Overall, these findings confirm that essential oils behave as complex bioactive systems in which chemical profiles govern biological performance, providing a solid basis for future structure–activity investigations and supporting their potential applications in natural pest management, nutraceutical development, and pharmaceutical research.

## Figures and Tables

**Table 1:** Chemical composition of *Asteriscus graveolens* essential oils aerial parts [33].

No ^a^	Compound	RI ^b^ Appl.	RI ^c^ Lit.	% ^d^
1	α-Pinene	933	1004	1.2
2	6-Methylhept-5-en-2-one	980	1302	0.3
3	Myrcene	982	1145	-
4	1,8-Cineole	1023	1183	0.1
5	Linalool	1081	1544	0.2
6	α-Thujone	1090	1384	-
7	α-Campholenal	1108	1446	-
8	Camphor	1124	1470	0.6
9	cis-Chrysanthenol	1146	1445	-
10	Albene	1152	1270	-
11	Terpinen-4-ol	1162	1580	0.1
12	α-Terpineol	1170	1700	0.2
13	Estragole	1177	1650	0.2
14	Carvotanacetone	1218	1664	0.2
15	cis-Chrysanthenyl acetate	1248	1526	2.1
16	Carvacrol	1279	2135	0.9
17	α-Copaene	1384	1476	-
18	Longifolene	1414	1562	-
19	trans-Caryophyllene	1418	1579	-
20	trans-α-Bergamotene	1431	1572	-
21	Sesquisabinene	1435	1642	-
22	epi-β-Santalene	1442	1629	-
23	α-Humulene	1452	1628	0.3
24	Alloaromadendrene	1458	1620	-
25	γ-Muurolene	1470	1680	-
26	Germacrene D	1474	1700	-
27	β-Bisabolene	1502	1754	-
28	γ-Cadinene	1504	1737	0.2
29	δ-Cadinene	1518	1705	0.1
30	(Z)-Nerolidol	1522	1518	0.4
31	Kessane	1528	1715	0.5
32	α-Cadinene	1530	1731	-
33	6-Oxocyclonerolidol	1555	1969	72.5
34	cis-8-Acetoxychrysanthenyl acetate	1564	2122	2.1
35	Caryophyllene oxide	1576	1921	0.1
36	Humulene epoxide II	1598	1975	1.3
37	6-Hydroxycyclonerolidol	1622	2246	9.9
38	τ-Cadinol	1631	2102	0.3
39	β-Eudesmol	1636	2160	0.2
40	α-Cadinol	1639	2221	-
41	Intermodeol	1643	2160	2.5
42	α-Oxobisabolene	1707	2266	0.2
Monoterpene hydrocarbons		1.5		
Oxygenated monoterpenes		4.6		
Sesquiterpene hydrocarbons		0.6		
Oxygenated sesquiterpenes		90		
Total identified		96.7		

^a^ The order of elution on an apolar column is given (Rtx-1). ^b^ Retention indices on the apolar column (Rtx-1). ^c^ Retention indices on an apolar column reported in the literature. ^d^ Relative percentages of components (%) are calculated for GC peak areas on the apolar column (Rtx-1), except for components with identical RIa (concentrations on the polar column are given). Note: -, not detected.

**Table 2:** Chemical composition of *Teucrium luteum* subsp. *flavovirens* essential oils aerial parts [34].

No ^a^	Compound	lRIa ^b^	Ria ^c^	Rip ^d^	% ^e^
1	α-Pinene	936	931	1017	12.0
2	1-Octen-3-ol	962	959	1442	0.3
3	Sabinene	973	963	1113	0.5
4	β-Pinene	978	969	1105	5.7
5	Myrcene	987	978	1149	0.2
6	p-Cymene	1015	1008	1256	0.2
7	1,8-Cineole	1024	1017	1202	0.1
8	Limonene	1025	1017	1189	1.4
9	Linalool	1086	1077	1544	0.9
10	α-Thujone	1089	1079	1411	0.1
11	1-Octen-3-yl acetate	1093	1085	1369	-
12	β-Thujone	1103	1090	1430	0.1
13	α-Campholenal	1105	1097	1479	0.1
14	Nopinone	1111	1101	1565	0.3
15	Camphor	1123	1113	1504	0.9
16	trans-Pinocarveol	1126	1116	1634	0.3
17	cis-Verbenol	1132	1121	1666	0.2
18	Menthone	1136	1125	1453	-
19	Pinocarvone	1137	1131	1556	0.1
20	Borneol	1150	1142	1670	0.2
21	Terpinen-4-ol	1164	1154	1595	0.6
22	Myrtenal	1172	1162	1615	0.5
23	α-Terpineol	1176	1165	1688	0.3
24	Myrtenol	1178	1172	1777	0.3
25	Verbenone	1183	1173	1670	0.6
26	trans-Carveol	1200	1191	1818	0.2
27	Carvone	1214	1209	1710	0.2
28	Carvotanacetone	1220	1214	1658	0.2
29	Geraniol	1235	1228	1832	-
30	cis-Chrysanthenyl acetate	1248	1236	1565	-
31	Bornyl acetate	1270	1262	1573	0.4
32	Carvacrol	1278	1275	2180	0.4
33	Myrtenyl acetate	1313	1300	1662	-
34	α-Terpinyl acetate	1335	1327	1688	0.4
35	α-Copaene	1379	1370	1484	-
36	β-Bourbonene	1386	1378	1511	0.9
37	β-Elemene	1389	1383	1584	-
38	trans-Caryophyllene	1421	1413	1589	7.0
39	γ-Elemene	1429	1424	1630	0.6
40	α-Humulene	1455	1446	1645	6.4
41	Dehydrosesquicineole	1466	1455	1708	1.1
42	Germacrene D	1479	1472	1697	1.0
43	β-Selinene	1486	1478	1705	2.2
44	cis-β-Guaiene	1488	1482	1762	0.2
45	7-epi-Cubebol	1490	1484	1870	0.7
46	Bicyclogermacrene	1494	1487	1718	1.6
47	Cubebol	1514	1503	1920	0.2
48	7-epi-α-Selinene	1519	1509	1678	3.6
49	δ-Cadinene	1526	1511	1744	0.1
50	Elemol	1541	1533	2058	16.4
51	E-Nerolidol	1553	1552	2027	1.7
52	Caryophyllene oxide	1578	1567	1957	2.2
53	Humulene epoxide II	1602	1592	2010	1.6
54	epi-Cubenol	1623	1612	2030	0.3
55	γ-Eudesmol	1618	1616	2189	5.2
56	τ-Cadinol	1633	1624	2141	0.2
57	τ-Muurolol	1633	1624	2158	0.2
58	β-Eudesmol	1641	1633	2190	4.3
59	Valerianol	1647	1637	2184	4.9
60	α-Eudesmol	1653	1637	2197	4.8
61	Bulnesol	1665	1640	2170	-
62	α-Bisabolol	1673	1650	2184	2.3
63	α-Cyperone	1741	1723	2307	0.3
Oxygenated monoterpenes		7.5			
Monoterpene hydrocarbons		20.2			
Oxygenated sesquiterpenes		48.3			
Sesquiterpene hydrocarbons		22.1			
Total identified		98.1			

^a^ Order of elution is given on an apolar column (Rtx-1). lRIa ^b^ = retention indices in the literature. Ria ^c^ = retention indices on the apolar column (Rtx-1). Rip ^d^ = retention indices on the polar column (Rtx-Wax). ^e^ Relative percentages of components (%) are calculated on GC peak areas on the apolar column (Rtx-1) except for components with identical RIa (concentrations are given on the polar column). Note: -, not detected.

**Table 3:** Mineral element composition of the studied species (mg/kg ± SD).

	*C. eriolepis*		*A. graveolens*		*T. luteum* subsp. *flavovirens*	
	Aerial Part		Aerial Part		Aerial Part	
	Mean	S.D.	Mean	S.D.	Mean	S.D.
Cr	0.64	0.02	0.56	0.02	0.56	0.01
Fe	20.18	1.12	25.44	1.22	56.31	1.31
F	11.50	0.50	6.11	0.52	9.41	0.51
I	4.00	0.54	2.10	0.41	4.45	0.44
Cu	0.08	0.01	0.08	0.01	0.06	0.01
Mn	0.50	0.02	0.44	0.02	0.41	0.02
Mo	9.28	0.91	7.70	0.88	9.94	0.90
Ni	0.37	0.01	0.32	0.01	0.21	0.01
Se	0.06	0.01	0.08	0.01	0.07	0.01
V	1.50	0.03	1.13	0.03	1.80	0.03
Zn	0.21	0.02	0.41	0.02	0.54	0.02
Sn	0.07	0.01	0.06	0.01	0.04	0.01
Co	0.65	0.01	0.40	0.01	0.52	0.01
K	155.12	4.12	177.65	4.22	233.88	4.71
Mg	37.57	1.12	44.12	1.15	67.07	2.12
Ca	194.97	6.61	150.09	7.76	244.71	9.81
Cl	16.78	0.74	21.44	1.41	17.11	0.94
S	27.41	1.66	33.40	2.01	36.76	2.13
Na	10.80	0.61	9.23	0.57	13.55	0.80
P	33.90	2.56	40.08	2.72	39.50	3.33
Al	19.20	1.10	15.40	2.30	21.45	2.25
Pb	0.10	0.03	0.15	0.04	0.18	0.02
Si	0.90	0.02	1.20	0.04	1.85	0.03

**Table 4:** Acaricidal activities (LC_50_ and LC_90_) of the studied essential oils against *T. urticae* adults after 48 h of treatment.

Essential Oil	T (h)	LC_50_ (µL/mL) (95% LD)	LC_90_ (µL/mL) (95% LD)	χ^2^	DF
*T. luteum* subsp. *flavovirens*	48	1.482 (0.979–2.621)	9.901 (8.181–12.131)	93.041	23
*A. graveolens*	48	2.843 (2.132–3.918)	15.983 (9.713–30.196)	87.460	23
*C. eriolepis*	48	0.539 (0.191–1.532)	6.121 (4.989–8.323)	117.118	23

**Table 5:** IC_50_ values (µg/mL) of essential oils determined by DPPH, FRAP, and β-carotene bleaching assays.

	DPPH (IC_50_ µg/mL)	FRAP (IC_50_ µg/mL)	β-Carotene Bleaching (IC_50_ µg/mL)
*C. eriolepis*	155.48 ± 4.83 ^e^ *	74.97 ± 4.19 ^d^	42.88 ± 2.43 ^a^
*A. graveolens*	124.68 ± 3.76 ^d^	60.93 ± 4.41 ^c^	77.47 ± 2.40 ^c^
*T. luteum* subsp. *flavovirens*	51.60 ± 1.87 ^b^	35.53 ± 1.05 ^b^	121.50 ± 9.69 ^d^
Ascorbic acid	23.60 ± 2.85 ^a^	12.12 ± 1.50 ^a^	54.57 ± 2.25 ^b^
BHT	104.73 ± 2.78 ^c^	34.22 ± 3.71 ^b^	77.93 ± 2.50 ^c^

* All data are expressed as mean ± SE (*n* = 3). Significant differences among values in the same column are indicated by different letters (*p* < 0.05).

**Table 6:** In vitro enzyme inhibition profiles of essential oils from *C. eriolepis, A. graveolens*, and *T. luteum* subsp. *flavovirens*.

Plant	Doses (mg/mL)	AChE Inhibition (%)	Tyrosinase Inhibition (%)	α-Glucosidase Inhibition (%)
*C. eriolepis*	0.2	48.63 ± 1.33 ^a^ *	45.23 ± 1.66 ^a^	59.00 ± 1.28 ^b^
	0.4	64.37 ± 1.72 ^b^	56.87 ± 1.45 ^b^	66.33 ± 0.85 ^c^
	0.8	81.77 ± 1.03 ^c^	66.53 ± 1.12 ^c^	76.30 ± 0.90 ^d^
	1.2	97.33 ± 1.53 ^e^	87.43 ± 1.11 ^e^	89.62 ± 0.42 ^e^
	Positive control	78.33 ± 1.78 ^c^	76.73 ± 1.79 ^d^	77.07 ± 1.65 ^d^
*A. graveolens*	0.2	46.43 ± 1.01 ^a^	50.23 ± 0.91 ^a^	46.05 ± 1.39 ^a^
	0.4	64.60 ± 1.64 ^b^	57.67 ± 0.68 ^b^	58.17 ± 1.00 ^b^
	0.8	80.67 ± 0.55 ^c^	71.53 ± 0.76 ^c^	67.50 ± 0.93 ^c^
	1.2	93.50 ± 0.85 ^d^	85.63 ± 1.06 ^d^	86.87 ± 0.46 ^e^
	Positive control	80.57 ± 1.17 ^c^	86.27 ± 0.96 ^d^	87.34 ± 0.83 ^e^
*T. luteum* subsp. *flavovirens*	0.2	56.30 ± 2.80 ^b^	48.53 ± 0.90 ^a^	46.60 ± 0.92 ^a^
	0.4	75.70 ± 1.86 ^c^	56.90 ± 0.20 ^b^	61.33 ± 1.56 ^b^
	0.8	85.97 ± 1.40 ^d^	67.00 ± 1.27 ^c^	65.73 ± 1.50 ^c^
	1.2	93.50 ± 1.20 ^d^	81.37 ± 1.00 ^d^	82.03 ± 1.64 ^d^
	Positive control	77.43 ± 1.05 ^c^	79.27 ± 1.06 ^d^	87.47 ± 0.70 ^e^

* Values represent mean ± SE; different letters indicate significant differences (*p* < 0.05).

**Table 7:** Collection locations, sampling periods, and essential oil yields of the studied plant species.

Species	Harvesting Site	Collection Time	GPS Coordinates	Voucher Specimen	Altitude (m)	Oil Yield (% (*w*/*w*))
*Cladanthus eriolepis*	Igoudmane (Errachidia)	May 2019	31°69′35.1″ N 5°31′95.3″ W	ER-19-15	1373	0.60 ± 0.09
*Asteriscus graveolens*	Merroutcha (Errachidia)	April 2017	31°33′14.7″ N 4°53′08.9″ W	ER-17-13	1009	0.62 ± 0.10
*Teucrium luteum* subsp. *flavovirens*	Errachidia	April 2016	31°55′42.2″ N 4°24′36.1″ W	ER-2016	1040	0.75 ± 0.10

## Data Availability

The original contributions presented in the study are included in the article; further inquiries can be directed to the corresponding authors.
